# Asprosin—A Fasting-Induced, Glucogenic, and Orexigenic Adipokine as a New Promising Player. Will It Be a New Factor in the Treatment of Obesity, Diabetes, or Infertility? A Review of the Literature

**DOI:** 10.3390/nu13020620

**Published:** 2021-02-14

**Authors:** Agnieszka Irena Mazur-Bialy

**Affiliations:** Department of Biomechanics and Kinesiology, Faculty of Health Science, Institute of Physiotherapy, Jagiellonian University Medical College, Skawińska 8, 31-066 Krakow, Poland; agnieszka.mazur@uj.edu.pl; Tel.: +48-12-421-9351

**Keywords:** asprosin, insulin resistance, obesity, appetite, fertility, PCOS, exercise

## Abstract

Asprosin is a recently discovered protein released during fasting conditions mainly by adipocytes from white adipose tissue. As a glucogenic peptide, it stimulates the release of glucose from hepatic cells by binding to the OLFR734 receptor and leading to the activation of the G protein-cAMP-PKA pathway. As it crosses the blood–brain barrier, it also acts as an orexigenic peptide that stimulates food intake through activation of AgRP neurons in the hypothalamus; thus, asprosin participates in maintaining the body’s energy homeostasis. Moreover, studies have shown that asprosin levels are pathologically elevated in obesity and related diseases. However, the administration of anti-asprosin antibodies can both normalize its concentration and reduce food intake in obese mice, which makes it an interesting factor to combat obesity and related diseases. Current research also draws attention to the relationship between asprosin and fertility, especially in men. Asprosin improves age- and obesity-related decrease in fertility potential by improving sperm motility. It should also be mentioned that plasma asprosin levels can be differentially modulated by physical activity; intense anaerobic exercise increases asprosin level, while aerobic exercise decreases it. However, further research is necessary to confirm the exact mechanisms of asprosin activity and its potential as a therapeutic target.

## 1. Introduction

It has long been known that adipose tissue is not only a store of fat reserves but also an extremely active endocrine organ. As a source of adipokines, it plays a role in the regulation of numerous physiological and pathological processes (for reviews, see [1,2]). Numerous adipokines show multifunctional, pleiotropic action [3], for example, irisin (for a review, see [4]), leptin [5], adiponectin [6] or adipsin, resistin, and visfatin [1]. In recent years, research studies have described another multifunctional adipokine known as asprosin, which seems to be a promising factor to combat obesity. This study aimed to conduct a narrative review of the literature by collecting current knowledge on asprosin—the newly discovered adipokine. The purpose of this review was to describe both the discovery of asprosin and the mechanisms of its action identified thus far as well as areas of current research on its potential clinical applications.

## 2. Materials and Methods

The review was conducted by searching relevant studies published in Web of Science and PubMed databases from the beginning of the discovery of asprosin, i.e., from 2016 to 7 February 2021, by using “asprosin” as the search keyword. The review included studies on the discovery of asprosin and articles describing the mechanisms of its action and those assessing changes in its level in response to the effects of various factors and physiological and disease states. Only articles published in English between 2016 and 2021 (7 February 2021) qualified for the review. The exclusion criteria were as follows: language of publication other than English and nonavailability of the full-text version of the article. Letters to the editor and summaries of conference speeches were excluded from the review. Studies in which there was no separate group for a real assessment of the action of asprosin were excluded. The review was prepared following the PRISMA (Preferred Reporting Items for Systematic Reviews and Meta-Analyses) guidelines.

## 3. Results

**Figure 1:**
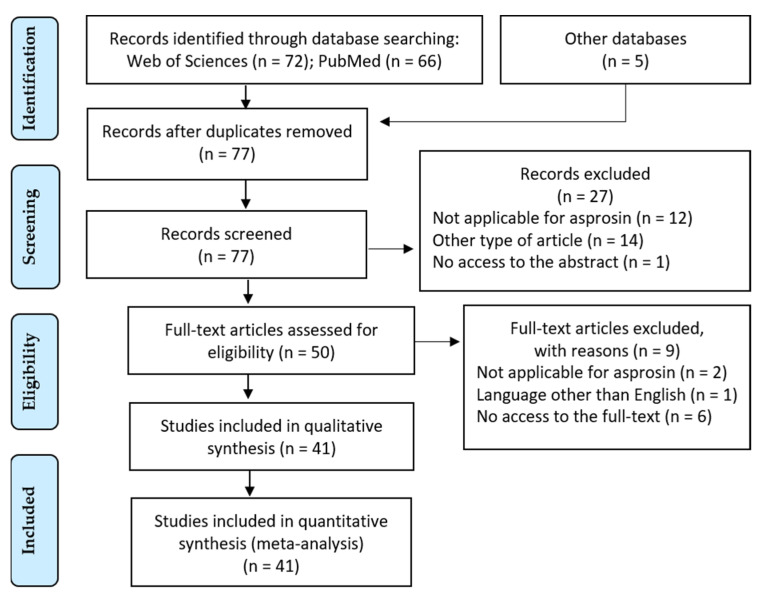
Diagram PRISMA—presents the different phases of the systematic review carried out.

A total of 143 articles were retrieved following the search in the PubMed and Web of Science databases. After removing duplicates, 77 articles remained. On the basis of the inclusion and exclusion criteria, 41 articles qualified for review. The PRISMA diagram (Figure 1) shows the individual stages of the review. The diagram shows the reasons for the exclusion of the articles and the final number of articles included in the analysis. After analyzing the articles that qualified for review, they were classified into thematic threads and described in the following subsections. 

## 4. Asprosin Discovery and Structure

Asprosin is a fasting-induced glucogenic hormone that was discovered and first described by Romere et al. in 2016 [7]. As a small 30 kDa protein with 140 amino acids (aa), it has three potential sites for N-glycosylation (the bacterial recombinant form of this protein is nonglycosylated and 17 kDa in size). Asprosin is encoded by two exons of the FBN1 gene (exon 65:11 aa; exon 66:129 aa), which also encodes profibrillin, and is formed by cleavage of the C-terminus of the fibrillin-1 protein [7,8]. The discovery of asprosin was supported by research on patients with rare mutation-induced neonatal progeroid syndrome (NPS), who are characterized by extreme thinness, lipodystrophy, and low calorie consumption and energy expenditure with a simultaneously lowered insulin level and euglycemia confirming high insulin sensitivity [7,8]. Romere et al. showed that patients with NPS exhibit a mutation in the FBN1 gene, which results in a significant, extreme reduction in the level of asprosin released by heterozygotes [7]. These observations were later confirmed in the murine [9] and rabbit Fbn1NPS/+ models [10], where it was found that the Fbn1NPS/+ phenotype protects the animals against the development of diet-induced obesity and diabetes mellitus (DM) [9]. Decreased levels of asprosin have also been noticed in patients with acromegaly, a syndrome often characterized by insulin resistance and diabetes, and adipose tissue dysfunction with reduced fat mass [11]. Asprosin is mainly produced and secreted by adipocytes from white adipose tissue during starvation [7], and its concentration in serum from healthy individuals reaches values in the range of 5.94 ± 3.04 nmol/L in men and 4.02 ± 0.49 nmol/L in women [12]. The half-life of asprosin in circulation is relatively short, approximately 20 and 145 min for the bacterial recombinant His-Tag form and the glycosylated form, respectively [9].

## 5. Glucogenic Action of Asprosin

**Figure 2:**
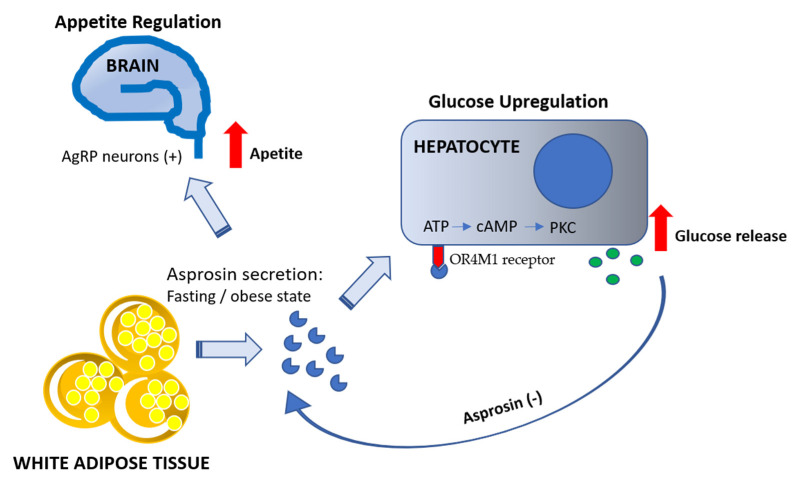
Mechanisms of asprosin action in fasting and obese state.

The main target organ of asprosin action is the liver, where it promotes glucose production and release [7,9]. As reported by Li et al. [13], asprosin acts through the Olfr734 receptor and induces glucose production in the liver in both fasting and obesity states. Olfr734 is a mouse ortholog of human OR4M1 receptor [8]. Asprosin, as a fasting-induced gluconeogenic hormone, uses a G protein and cyclic AMP (cAMP) messenger system for the activation of protein kinase A (PKA) in the liver and increases the release of glucose from hepatocytes [7]. Moreover, as reported by Romere et al., asprosin action is independent of the activation of the glucagon and catecholamine axis [7], which is also involved in glucose release [14] (Figure 2). High insulin levels reverse the action of asprosin by inhibiting asprosin-mediated increase in PKA activity and glucose release [7]. On the basis of these findings, asprosin seems to be in a functional opposition to insulin. Moreover, asprosin levels are strongly correlated with glucose levels, as low glucose levels stimulate asprosin production (fasting condition), while high levels inhibit it (feeding condition). Asprosin level fluctuates according to the circadian rhythm; after overnight fasting, its level increases significantly in humans, mice, and rats and then decreases after a meal. An injection of recombinant asprosin also leads to an immediate glucose peak and hyperinsulinemia [7].

## 6. Asprosin and Appetite

Asprosin is an important hormone in appetite regulation. As reported by Duerrschmid et al. [9], asprosin crosses the blood–brain barrier and directly affects appetite stimulation by activating orexigenic agouti-related peptide (AgRP) neurons in the hypothalamus, simultaneously leading to the indirect inhibition of the arcuate proopiomelanocortin (POMC) anorexigenic neurons. It has been shown that the effect of asprosin is associated with the activation of the Gαs–cAMP–PKA axis. AgRP+ neurons are crucial for regulating food intake and energy homeostasis [15]. This small subpopulation of the arcuate nucleus neurons releases a highly orexigenic neuropeptide AgRP as well as neuropeptide Y (NPY) and GABA transmitters that are important for feeding promotion [16]. Research has shown that mutation in the FBN1 gene (Fbn1NPS/+ phenotype) induces physiological changes in the activity of AgRP+ neurons in mice (lower firing rate and membrane potential); this condition was reversed after intracerebroventricular injection of asprosin [9]. Moreover, the administration of asprosin reversed the hypophagy observed in mice bearing the FBN1 gene mutation [9], which is also observed in patients with NPS. It is believed that asprosin acts directly on AgRP+ neurons, causing their slow and gradual activation, which translates into a gradual increase in appetite and food consumption. This mode of interaction is more similar to the action of leptin than of ghrelin, which causes a sudden and rapid increase in appetite [17]. In this regard, asprosin as a fasting-induced hormone stimulates food intake and participates in maintaining the body’s energy balance under physiological conditions. A different situation is observed in the case of obesity and insulin resistance, where asprosin levels are pathologically elevated, which, in turn, increases appetite and disturbs the maintenance of energy homeostasis. Hence, the study conducted by Duerrschmid et al. [9], which demonstrated the effectiveness of using anti-asprosin antibodies in suppressing food intake by insulin-resistant obese mice, is particularly valuable. Administration of anti-asprosin antibodies significantly reduced pathologically elevated asprosin level and decreased AgRP neuron activity [9]. It should also be mentioned that researchers have shown that an increased level of asprosin may accompany anorexia nervosa and may be involved in the development of bulimia in these patients [18]. Other authors speculate that a decreased level of asprosin observed in oncological patients is either involved in the development of cancer anorexia or could be used to combat this condition [19]. On the basis of these findings, asprosin seems to be a promising target for treating obesity and related diseases; however, further research is necessary to confirm this possibility.

## 7. Asprosin and Insulin Resistance, Diabetes, and Obesity

As reported by Romere et al. [7], in the state of insulin resistance, a compensatory model with a high level of asprosin and insulin was observed, but the use of specific anti-asprosin antibodies normalized the abovementioned effect. Pathologically elevated asprosin level was also noted in patients with obesity [20,21], insulin resistance [22,23], and diabetes mellitus type 1 (DM1; [24]) and type 2 (DM2; [25,26,27]), while this was reduced in mice with streptozocin-induced diabetes [28]. Therefore, research on the possibility of using anti-asprosin antibodies as well as assessing their effectiveness seems to be particularly important in the case of an increase in the number of patients with obesity and related diseases. As reported by Zhang et al. [25], elevated asprosin levels are a risk factor for the development of DM2; in addition, patients with DM2 develop abnormal release of asprosin in response to changes in glucose levels [26]. Moreover, elevated levels of asprosin observed in DM2 patients correlate not only with insulin resistance, but also with the atherosclerotic risk factor of cardiovascular diseases [27]. Considering the prodiabetogenic effect of asprosin [7], it is important to note that asprosin release may also be induced by hyperlipidemia, and through the activation of the TLR4/JNK-mediated pathway, it may lead to pancreatic beta-cell dysfunction and consequently, impairment of insulin release [29]. Moreover, recombinant asprosin intensifies the inflammatory response in a dose-dependent manner [29]. Asprosin also impairs muscle cell sensitivity to insulin by promoting inflammation and endoplasmic reticulum (ER) stress, leading to skeletal muscle insulin resistance, which depends on the activation of the PKCδ/SERCA-2 pathway by asprosin [30]. Moreover, Deng et al. [31] noticed that the level of asprosin independently correlates with the urinary albumin/creatinine ratio, which is considered an index helpful in the detection of early diabetic nephropathy, and in combination with adiponectin level—asprosin could be a marker of early NAFLD diagnosis [32]. Furthermore, Zhong et al. [33] noted that the level of asprosin increases in pregnant women with gestational diabetes mellitus (GDM) and their newborns, which, as suggested, may be a marker of early GDM diagnosis in this group. Baykus et al. [34] revealed that the level of asprosin is elevated not only in pregnant women with GDM but also in women with pre-eclampsia. Moreover, in women with intrauterine growth restriction, a significantly lower level of asprosin was observed than in healthy pregnant women. Asprosin expression was also observed in the placenta, but no significant correlations were found [33]. Asprosin level is also elevated in obese children more significantly than in overweight children, with girls having higher levels than boys [35]. The current review of the literature in this area has been prepared by Janoschek et al. [36]. On the basis of these findings, asprosin seems to be a factor favoring both the development of obesity and accompanying diseases such as DM2, while promoting the development of insulin resistance. Promising results of research on the use of anti-asprosin antibodies indicate the use of these antibodies as a potential therapeutic tool in pathological conditions involving asprosin.

## 8. Asprosin in Myocardial and Microvascular Damage

Zhang et al. [37] have shown asprosin as a promising cardioprotective agent. Asprosin regulates the function and survival of mesenchymal stromal cells (MSCs) and has a positive effect on the effectiveness of their use in myocardial infarction (MI) treatment [37]. Asprosin pretreatment increases targeting of MSCs to the site of the lesion—here, MI. Moreover, intracardiac administration of MSCs pre-incubated with asprosin improves the infarcted cardiac ejection and reduces cardiac fibrosis. In addition, asprosin reduces free radical-induced MSCs damage and apoptosis by activating the ERK1/2 and PI3K/AKT pathways, upregulating the expression of the SOD-2 antioxidative enzyme, and inhibiting further free radical production. The cardioprotective effect of asprosin was also shown by Wen et al. [38]. They proved, in a cellular model, that asprosin protects cardiomyocytes through hypoxia-induced apoptosis. However, in clinical trials, they have shown that patients with dilated cardiomyopathy (DCM) with a high level of asprosin have a lower risk of adverse clinical outcomes than patients with lower levels of asprosin (<210 ng/mL). In other studies, Chen et al. [39] showed that asprosin has a protective effect against damage of cardiac microvascular endothelial cells caused by high glucose concentration. Considering the above, it can be concluded that asprosin seems to be a promising cardioprotective and cytoprotective factor protecting cells against damage related to hypoxia or the action of free radicals. However, further research is necessary to understand the exact mechanism of its protective action.

## 9. Asprosin and Male/Female Fertility

As reported by Wei et al. [40], asprosin and its interaction with OLFR734 affects male fertility, especially in obese individuals. OLFR734 is highly expressed in the testis [41] and in a smaller amount in the ovary [40]. The effect of obesity on the hypothalamic–pituitary–gonadal axis is well described [42,43]. It is known that obesity reduces the ability of the testes to produce the correct amount of testosterone and also reduces the effective number of spermatozoa produced. In studies conducted on Olfr734 knockout mice, Wei et al. have shown that disabling of the OLFR734 gene does not affect sperm viability and morphology but significantly reduces their progressive motility, which is important for sperm movement in the female genital tract. Moreover, the authors observed fertility reduction in OLFR734-/- males measured by the number of offspring as compared to that in wild-type mice [40]. On the other hand, asprosin treatment significantly improved progressive sperm motility with a positive impact on fertility potential [40]. In addition, the well-known decrease in age-related sperm motility [42,43] by administering asprosin resulted in an increase in ATP levels, and cGMP reversed the age-related decrease in fertility by improving motility. A similar effect was also observed by the authors in relation to the decrease in fertility associated with obesity. An improvement in progressive motility and consistently improved fertility were noted [40].

The role of asprosin and OLFR734 in ovary function and female fertility is currently unclear. However, Maylem et al. [44] suggest that asprosin may be an important factor that regulates the function of ovarian follicles. The expression of both the asprosin precursor, namely FBN1, and its putative OR4M1 receptor (olfactory receptor family 4 subfamily M member 1), significantly varies between large and small granulosa and theca cells. Moreover, asprosin increased the LH-induced production of androstenedione without affecting the production of progesterone. It is also worth mentioning the research of Leonard et al. [45] who showed that the level of asprosin changes in the course of a woman’s menstrual cycle and that the use of contraception is accompanied by a decrease in the level of asprosin. Nevertheless, further studies are necessary to verify the role of asprosin in the functioning of the ovaries and in female fertility.

## 10. Asprosin and Polycystic Ovary Syndrome

Polycystic ovary syndrome (PCOS) is a common disease that affects nearly 6–10% of women of childbearing age; anovulation and hyperandrogenism are observed during the course of this disease, resulting in infertility disorders [46]. The role of asprosin in the pathogenesis and progression of PCOS is currently unclear. Research conducted by Alan et al. [23] showed a significant relationship between the elevated level of serum asprosin and the risk of PCOS development in women with insulin resistance. Moreover, Li et al. [41] indicated that asprosin level is elevated in women with PCOS and is positively correlated with the level of testosterone and prolactin but negatively correlated with the levels of estradiol and sex hormone-binding globulin (SHBG). In contrast, studies conducted by Chang et al. [47] showed that asprosin levels do not increase significantly in women with PCOS.

## 11. Physical Activity and Asprosin Production

As reported by Romere et al. [7], the expression of the asprosin-encoding *FBN1* gene was found not only in adipose tissue but also in skeletal muscle. Several studies have indicated that the level of this protein is modulated by physical activity. Ko et al. [48] indicated that an 8-week aerobic exercise program on a treadmill effectively reduced the level of asprosin in type 1 diabetes mellitus mice. The authors observed that asprosin-dependent hepatic PKA/TGF-β pathways were affected by a reduction in PKA and TGF-β levels as well as by an increase in AMPK downstream pathways. Nakhaei et al. [49] also showed that the asprosin level could be modulated by physical activity. The authors showed a significant decrease in asprosin levels in rats with metabolic syndrome after intermittent and continuous swimming training. The abovementioned studies suggest that properly selected physical activity may be a factor that could reduce the high level of asprosin observed during the course of diabetes or metabolic syndrome. However, further research is needed to determine the exact mechanisms of this phenomenon. Currently, no studies have verified the effect of various forms of physical activity in order to fully characterize the possibilities of modulating the level of asprosin in the event of its pathological overexpression; however, the available data indicate that physical activity has a great potential for use in this field. Studies conducted by Wiecek et al. [12] on healthy individuals are also interesting, which showed that short-term high-intensive anaerobic exercise—here, a 20 s sprint on a bicycle—significantly increases plasma asprosin levels in healthy women, but not in men. Moreover, asprosin level was positively corelated with adiponectin and irisin levels but negatively correlated with leptin level. The effect of acute aerobic exercise on the level of asprosin was also assessed by Ceylan et al. [50] in a group of healthy and obese men who participated in a 30-min training session in the morning or evening. The authors showed that a 30-min training session significantly reduces the level of asprosin, but this effect is more pronounced in the group of obese men if the training was held in the evening hours (8:00 P.M. to 10:00 P.M.). The readers can refer to Ceylon et al. [51] for a full review of the effect of exercise on asprosin release. The studies assessing the effect of whole-body cryotherapy on asprosin level in menopausal women are also noteworthy. The asprosin level decreased significantly in these women after 20 sessions, with a reduction in abdominal obesity [52]. These results suggest that both the duration and intensity of exercise may have a diverse, sometimes opposing, effect on asprosin release.

## 12. Research on the Potential of Asprosin as a Drug—Promising Research Directions

The majority of current research focuses on understanding the mechanisms of action of asprosin, the factors influencing its release, and the correlations between the level of asprosin and the occurrence of a given health condition or disease. As it is a relatively young molecule, few research teams have taken up the challenge of using asprosin as a therapeutic agent for treating selected diseases or conditions. Undoubtedly, the studies conducted thus far show the enormous potential of this molecule both in terms of its appropriate therapeutic effect and the possibility of using antibodies against asprosin in specific situations to reduce its level. In the first case, the potential of asprosin is assessed in the context of the possibility of stimulating the appetite and thus increasing body weight, which was reflected in the patent application [53]. In the research on combating obesity, the possibility of using antibodies directed against asprosin is also considered, which can enable lowering of its level and thus reduce the feeling of hunger in obese patients (see patents: [53,54]). Another noteworthy development is a patent application [55] aimed at assessing the effectiveness of asprosin albumin administration for treating ischemic heart damage, which has previously been confirmed in animal models. Considering the increasing number of reports on the mechanisms of action of asprosin and its broad functional connections, other interesting areas are likely to be the assessment of its application in supporting the fertility of both men and women.

## 13. Conclusions

Asprosin as a newly recognized adipokine seems to be an interesting subject for future research. As a fasting-induced hormone, it regulates food intake and energy supply through a gradual increase in appetite. Its level correlates with the content of adipose tissue, which is its main source. Therefore, its increased levels are observed in obese and overweight people. As a factor that increases appetite and induces glucose release, it seems to contribute to the development of obesity, metabolic syndrome, and diabetes. On the other hand, asprosin seems to be a promising target to combat obesity and metabolic diseases by using anti-asprosin antibodies. Asprosin seems to be a multifunctional molecule (Table 1), which is undoubtedly due to the location of its receptors in numerous tissues of our body, such as the liver, kidneys, heart, muscles, testes, and ovaries. Currently, knowledge on asprosin is limited, as probably many aspects of its physiological and pathophysiological activity remain to be discovered. There is, however, no doubt that it is already an interesting object of scientific inquiry.

## Figures and Tables

**Table 1:** Characteristics of asprosin classified into main thematic threads.

Thematic Threads	Description	References
Asprosin discovery	Asprosin structure	[7,8]
Asprosin action	Olfr734 as an asprosin receptor	[12]
	FBN1 gene mutation reduces asprosin production	[7,9,10]
	Starvation induces asprosin production by white adipose tissue	[7]
	Obesity state induces asprosin production by white adipose tissue	[13]
	Mechanism of asprosin action via cAMP/PKC activation	[7]
	Liver as a target organ for asprosin action—induction of glucose release	[7,9]
Appetite regulation	Asprosin activates orexigenic agouti-related peptide (AgRP) neurons leading to appetite stimulation by the Gαs-cAMP-PKA axis	[9]
	Anti-asprosin antibodies efficiently suppress AgRP neurons activation and food intake by insulin-resistant obese mice	[9]
Elevated asprosin level in diseases state	in patients with obesity	[20,21,35,36]
	in patients with insulin resistance	[22,23]
	in patients with diabetes mellitus type 1	[24]
	in patients with diabetes mellitus type 2	[25,26,27]
	in patients with anorexia nervosa	[18]
	in pregnant women with gestational diabetes mellitus	[33]
	in pregnant women with pre-eclampsia	[34]
Decreased asprosin level in diseases state	patients with neonatal progeroid syndrome (NPS)	[7,8]
	in oncological patients with anorexia	[19]
	in women with intrauterine growth restriction	[34]
	in patients with acromegaly	[11]
	In mice with streptozocin-induces diabetes	[28]
Pathophysiology of asprosin action	Asprosin induces pancreatic beta-cell dysfunction and impairs insulin release	[29]
	Asprosin impairs muscle cell sensitivity to insulin	[30]
Cardioprotection	Asprosin improves the effectiveness of mesenchymal stromal cells (MSCs) use in myocardial infarction (MI) treatment	[37]
	Asprosin protects cardiomyocytes through hypoxia-induced apoptosis	[38]
	Asprosin protects cardiac microvascular endothelial cells against damage caused by high glucose concentration	[39]
Fertility	Asprosin improves progressive sperm motility and fertility potential	[40]
	Asprosin regulates the function of ovarian follicles	[44]
	Asprosin level changes in the course of menstrual cycle	[45]
Physical activity in asprosin reduction	8-week aerobic exercise program reduces asprosin in mice with type 1 diabetes mellitus	[48]
	Intermittent and continuous swimming training decrease asprosin levels in rats with metabolic syndrome	[49]
	Acute aerobic exercise reduces asprosin level in healthy and obese men	[50]
	Short-term high-intensive anaerobic exercise increases asprosin level in healthy women	[12]
	Whole-body cryotherapy reduces asprosin level in menopausal women	[52]
